# Gemcitabine-oxaliplatin combination for ovarian cancer resistant to taxane-platinum treatment: a phase II study from the GINECO group

**DOI:** 10.1038/sj.bjc.6604878

**Published:** 2009-02-03

**Authors:** I Ray-Coquard, B Weber, J Cretin, Z Haddad-Guichard, E Lévy, A C Hardy-Bessard, M C Gouttebel, J-F Geay, A Aleba, H Orfeuvre, C Agostini, J Provencal, J M Ferrero, D Fric, N Dohollou, D Paraiso, J Salvat, É Pujade-Lauraine

**Affiliations:** 1 Centre Léon Bérard, 28 rue Laennec, 69008 Lyon, France; 2 Centre Alexis Vautrin, 6 Avenue de Bourgogne, 54511 Vandœuvre-lès-Nancy Cedex, France; 3 Clinique Bonnefon, 45 Avenue Carnot, 30100 Alès, France; 4 Centre Hospitalier William Morey, 7 quai de l'hôpital, 71321 Chalon-sur-Saône Cedex, France; 5 Hôpital Européen Georges Pompidou, 20 rue Leblanc, 75015 Paris, France; 6 Clinique Armoricaine de Radiologie, 21 Rue du Vieux Séminaire, 22015 Saint Brieuc Cedex, France; 7 Centre Hospitalier, 179 Boulevard Maréchal Juin, 26953 Valence Cedex 9, France; 8 Hôpital André Mignot, 177 rue de Versailles, 78157 Le Chesnay Cedex, France; 9 Centre Hospitalier, 40 avenue Charles de Gaulle, 79021 Niort Cedex, France; 10 Centre Hospitalier Fleyriat, 900 route de Paris, BP 401, 01012 Bourg en Bresse, France; 11 Centre Hospitalier Général, BP1125, 73011 Chambéry Cedex, France; 12 Centre Hospitalier Général, BP 2333, 74011 Annecy, France; 13 Centre Antoine Lacassagne, 33 Av de Valombrose, 06189 Nice cedex 02, France; 14 Institut Privé de Cancérologie, 43 avenue Marie Reynoard, 38100 Grenoble, France; 15 Polyclinique Bordeaux Nord, 15 rue Claude Boucher, 33300 Bordeaux, France; 16 Centre Hospitalier de l'Agglomération Montargoise, 658 Rue des Bourgoins, 45207 Amilly Cedex, France; 17 Centre Hospitalier de Thonon-les-Bains, 3 avenue Dame, 74203 Thonon les Bains cedex, France; 18 Hôpital Hôtel-Dieu, 1 place du Parvis Notre-Dame, 75004 Paris, France

**Keywords:** combination chemotherapy, platinum-resistant, ovarian cancer, oxaliplatin, gemcitabine, toxicity

## Abstract

Advanced ovarian carcinoma in early progression (<6 months) (AOCEP) is considered resistant to most cytotoxic drugs. Gemcitabine (GE) and oxaliplatin (OXA) have shown single-agent activity in relapsed ovarian cancer. Their combination was tested in patients with AOCEP in phase II study. Fifty patients pre-treated with platinum–taxane received q3w administration of OXA (100 mg m^–2^, d1) and GE (1000 mg m^–2^, d1, d8, 100-min infusion). Patient characteristics were a : median age 64 years (range 46–79),and 1 (84%) or 2 (16%) earlier lines of treatment. Haematological toxicity included grade 3–4 neutropaenia (33%), anaemia (8%), and thrombocytopaenia (19%). Febrile neutropaenia occurred in 3%. Non-haematological toxicity included grade 2–3 nausea or vomiting (34%), grade 3 fatigue (25%),and grade 2 alopecia (24%). Eighteen (37%) patients experienced response. Median progression-free (PF) and overall survivals (OS) were 4.6 and 11.4 months, respectively. The OXA–GE combination has high activity and acceptable toxicity in AOCEP patients. A comparison of the doublet OXA–GE with single-agent treatment is warranted.

With platinum taxane-based chemotherapy and debulking surgery, 70–80% of patients with advanced ovarian cancer are free of clinical disease (Kerbrat *et al*, 2001). Although primary chemotherapy achieves high response rates, about 75% of patients will subsequently relapse with incurable disease (Lhomme *et al*, 2004). Relapsed patients can be classified into one of three categories, according to the therapy-free interval (TFI) between the end of platinum-based chemotherapy and relapse. Those with TFI >6 months generally have relatively chemo-sensitive tumours that may respond to platinum therapy (platinum-sensitive disease) (Markman and Hoskins, 1992). In contrast, patients with TFI <6 months or no response to initial therapy (chemo-resistant or -refractory disease) have an extremely poor outcome (Eisenhauer *et al*, 1997). The prognosis of patients who progress during first-line chemotherapy or who experience an early relapse (treatment-free interval <6 months) is extremely poor, with <5% long-term disease-free survival (Eisenhauer *et al*, 1997; Gordon *et al*, 2001). In these cases, standard treatment consists of single-agent regimens for palliation and disease control.

Oxaliplatin (OXA) is a diaminocyclohexane platinum derivative non-cross resistant to cisplatin. Oxaliplatin–DNA adducts are not recognised by the proteins of the mismatch repair system (Fink *et al*, 1997). This may explain the activity of OXA in platinum-resistant ovarian cancers (Fink *et al*, 1996; Raymond *et al*, 2002). In a randomised study of 86 relapsed ovarian cancer patients, OXA at a dose of 130 mg m^–2^ q3w has yielded median response rates and time to progression similar to those of paclitaxel (Piccart *et al*, 2000). Gemcitabine (GE) is an antimetabolite that inhibits DNA synthesis and blocks DNA repair pathways, a modulation that may be useful in overcoming platinum resistance.

The combination of OXA and GE is synergistic *in vitro* (Raymond *et al*, 2002). We hypothesised that this combination might provide more effective therapy for patients with relapsed ovarian cancer. The present multicentre phase II trial was designed to evaluate the efficacy and toxicity profile of OXA 100 mg m^–2^, d1, combined with GE 1000 mg m^–2^, d1 and d8, every 3 weeks in patience with advanced ovarian carcinoma in early progression (AOCEP).

## Patients and methods

### Objectives

The primary objective of the present study was to assess the anti-tumour activity of the OXA–GE combination in AOCEP patients. The main criterion for efficacy was objective response rate; secondary criteria were time to progression, response duration, and overall survival (OS). Our secondary objective was to determine the type, severity, and frequency of adverse events associated with OXA–GE treatment in these patients. The study design was an open, non-comparative, prospective phase II study.

The study was carried out according to good clinical practice guidelines, in accordance with the declaration of Helsinki, and was approved by local ethics committees. Approval was gained from local review boards, and written informed consent was obtained from each participant before inclusion. An independent monitoring institute was responsible for data control.

### Patients' eligibility

Eligibility criteria were: age >18 years; histologically confirmed diagnosis of advanced ovarian cancer; recurrent ovarian cancer treated with one or two earlier lines of platinum- and taxane-based chemotherapy, the last one consisting of a carboplatin–paclitaxel combination; measurable or evaluable disease documented by imaging according to the response evaluation criteria for solid tumours (RECIST), and blood CA 125 level >40 UI; progression of disease during first-line treatment, or treatment-free interval before inclusion <6 months; Eastern Cooperative Oncology Group (ECOG) performance status score <2; adequate haematological and organ functions; and written informed consent.

Exclusion criteria were: previous treatment with >2 lines of chemotherapy, previous total abdominal radiotherapy; brain or meningeal metastasis; grade >1 peripheral neuropathy according to the National Cancer Institute—Common Toxicity Criteria (NCI—CTC) version 2.0; severe cardiac dysfunction or uncontrolled hypertension; previous or concurrent malignancy other than ovarian cancer (with the exception of cutaneous basal cell carcinoma and cervical intraepithelial neoplasia); concurrent serious, uncontrolled medical (including bowel occlusion or subocclusion) or psychiatric disease; previous treatment with either OXA or GE; glomerular filtration rate calculated according to the Cockroft–Gault formula <60 ml min^–1^, total bilirubin concentration >1.25 × upper normal limit, liver transaminases >2.5 × upper normal limit, absolute neutrophil count <2.0 × 10^9^ l^–l^, and platelet count <100 × 10^9^ l^–l^.

### Treatment plan and drug administration

In all eligible patients treatment was administered through a central venous catheter: GE 1000 mg m^–2^ on days 1 and 8, administered over 100 min (10 mg m^–2^ min^–1^) after dilution in 250 ml normal saline, and OXA 100 mg m^–2^ on day 1, administered over 2 h 30 min after GE infusion after dilution in 250 ml of 5% glucose. All patients received standard antiemetic prophylaxis. Treatment was repeated every 21 days if blood counts returned to normal levels (neutrophil >1.5 × 10^9^ l^–l^ and platelets >100 × 10^9^ l^–l^) and non haematological toxicity resolved to grade <1.

This regimen was given for a minimum of two cycles in the absence of disease progression, unacceptable toxicity or patient refusal. An evaluation of response was performed after two courses to determine whether the treatment should be continued, then repeated every two cycles. After six courses, the patients could continue therapy for three further cycles if, in the opinion of the attending physician, further clinical benefit could be expected.

### Dose modifications

Treatment delays or dose modifications were decided based on NCI—CTC toxicity grading performed on each treatment day.

#### Haematological toxicity

Patients with neutropenic fever, grade 4 neutropaenia lasting >7 days or grade 4 thrombocytopaenia could continue treatment with a reduction of one dose level for each drug (OXA 85 mg m^–2^ on day 1 and GE 800 mg m^–2^ day^–1^ on days 1 and 8). The use of granulocyte-colony stimulating factor (G-CSF) was then authorised, at the physician's discretion.

In case of recovery to an absolute neutrophil count of ⩾1.5 × 10^9^ l^–l^ or more and a platelet count >100 × 10^9^ l^–l^in ⩽ 7 days, OXA and GE doses were reduced by one dose level. Any delay of >14 days in any course of treatment necessitated patient withdrawal from the study.

Day 8 of GE administration was suppressed if blood counts on that day showed an absolute neutrophil count of <0.5 × 10^9^ l^–l^ and a platelet count of <50 × 10^9^ l^–l^. Day 8 dose of GE was reduced to 600 mg m^–2^ if blood counts on that day showed an absolute neutrophil count between 0.5 × 10^9^ l^–l^ and 1.5 × 10^9^ l^–l^, and or a platelet count between 50 × 10^9^ l^–l^ and 100 × 10^9^ l^–l^.

#### Non-haematological toxicity

Oxaliplatin was reduced to 85 mg m^–2^ in case of grade 1 peripheral neurotoxicity, to 65 mg m^-2^ in case of grade 2, and stopped in case of grade 3. It was discontinued in patients with grade 3 hypersensitivity reaction. Gemcitabine was reduced to 800 mg m^–2^ in case of grade >2 mucositis.

Patients who required more than two dose reductions of the same drug were withdrawn from the study treatment. When dose reduction was required, no subsequent dose escalation was allowed.

### Evaluation of response and survival

Procedures for disease evaluation included standard physical examination and CA 125 level determination at each cycle, as well as computed tomography scan of the abdomen and pelvis and two-view chest X-ray every two courses. Objective responses were evaluated using the RECIST (Therasse *et al*, 2000). In the absence of measurable disease, serologic response was determined according to CA 125 level kinetics using GCIG criteria (Vergote *et al*, 2000).

Duration of response was measured from the time of initial documented response to the first sign of disease progression. Overall survival was evaluated by measuring the interval from the beginning of treatment to the date of last follow-up or date of death, whichever occurred first. Time to progression was defined as the time from the date of treatment to documentation of tumour progression.

### Determination of toxicity

Toxicity was evaluated using the NCI—CTC scale, version 2.0. All documented side effects were included, regardless of their relationship to study treatment. Haematological toxicity was evaluated weekly by complete blood count, whereas non-haematological toxicity was assessed before each treatment cycle. Chemotherapy was administered when the patient's neutrophil count was >1.5 × 10^9^ l^–l^, and the platelet count >100 × 10^9^ l^–l^.

### Criteria for withdrawal from the study

Patients were removed from the study for any of the following reasons: (i) evidence of progressive disease after a minimum of two cycles of therapy; (ii) development of unacceptable toxicity; (iii) patient's refusal or inability to comply with protocol requirements.

### Statistical analysis

A multi-stage phase II Fleming design was used to test whether the efficacy rate (response rate) was at least 20%, which we viewed as clinically promising, or at most 5%, which we viewed as not clinically promising (Fleming, 1982). With 45 evaluable patients, this trial had 90% power to detect an efficacy of 20% with a 0.05 level of significance. Interim analysis was performed after the first 15 and 30 patients. As we expected a 10% rate of ineligibility, the total number of patients planned for inclusion in the study was 50. The primary end-point of our study was objective response (CR plus PR). The response rate was calculated from all included patients based on the intention-to-treat principle, with determination of the corresponding 95% confidence interval (CI). Survival rates and time to progression were analysed by the Kaplan–Meier method using SPSS® version 10.0.

## Results

### Patient characteristics

From May 2001 to May 2002, 50 patients were enrolled into the study. Patient characteristics are described in detail in Table 1. The median age was 64 years (range 46–79). All patients received a platinum–taxane regimen as first-line treatment. A majority of them had received only one earlier line of chemotherapy. Of the eight patients treated in the third line, five had received earlier treatment with carboplatin alone, one with topotecan, one with carboplatin followed by anti-aromatases, and one with a combination of anthracyclins, paclitaxel and carboplatin. Overall, half of the patients had measurable disease, whereas 46% had no measurable disease but elevated levels of CA 125.

One patient, who did not receive any study treatment owing to rapid clinical deterioration due to bowel obstruction, was excluded from all statistical evaluations.

### Treatment

A median of six cycles (range, 1–9) was administered. Twenty-six (52%) of the 50 patients received the planned six cycles. The major cause for early discontinuation was disease progression in 17 patients, whereas seven patients did not complete the study because of adverse events (three patients), death without disease progression (two patients: one pulmonary embolism, one myocardial infarction), patient or physician decision (two patients).

The median dose of GE on day 1 remained close to 1000 mg m^–2^ for all 211 cycles administered. Of the 422 doses of GE planned, 37 (8.8%) were omitted (D8) and 57 (13.5%) were reduced, mainly because of neutropaenia, with a relative mean dose intensity of 85%. Of the 211 doses of OXA, none (0%) was omitted and 19 (9%) were reduced, mainly because of neurotoxicity. The median dose of OXA gradually decreased from 100 mg m^–2^ at cycle one to 96.8 mg m^–2^ at cycle six, with a relative mean dose intensity of 93%.

### Clinical response

**Figure 1:**
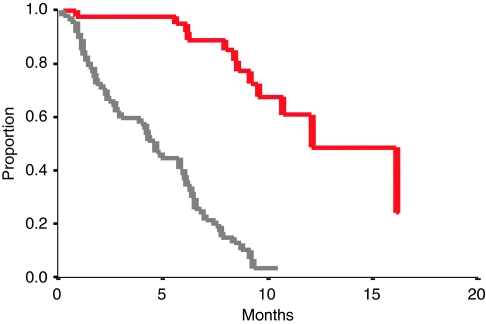
Progression–free survival (PFS) (light grey line) and overall survival (OS) (red line) for the all population (*n*=50). A full colour version of this figure is available at the *British Journal of Cancer* online.

The overall response rate at the end of treatment (six cycles) was 37% (95% CI, 24–52%) (Table 2). With a median follow-up of 16 months, the median progression-free survival (PFS) for the whole group was 4.6 months (range 0.3–10.4), and the median OS was 11.4 months (range 0.9–27) (Figure 1). The objective response rate in patients with measurable disease was 31% (*n*=26). In patients with only CA 125 assessable disease (*n*=23), the response rate evaluated according to CA 125 GCIG criteria was 43%. Interestingly, no PFS differences were seen according to the method of response evaluation: patients with partial or complete response evaluated with the clinical RECIST criteria had a PFS of 6.8 months *vs* 6.5 months for those who were evaluated with GCIG serological criteria. Patients with stable disease had a PFS of 4.9 and 4.2 months according to clinical and serological evaluation, respectively. Finally, progressive patients had a very poor median PFS of 1.4 months.

Although the trial was not designed to evaluate treatment activity in the different subsets of patients, we analysed response rates according to the treatment-free interval. For the five patients who had undergone progression under prevoius chemotherapy, no response to the GE–OXA combination was observed. The response rate was 44% (4/9 patients) for patients who relapsed between 0 and 3 months after previous therapy, and 42% (14/33 patients) for those who relapsed in the 3–6-month interval.

### Toxicity

A total of 211 chemotherapy cycles were administered to the 50 enrolled patients (median 6; range 1–8). Haematological side effects represented the main toxicity of the GE–OXA combination (Table 3). Blood transfusions were required in seven patients (12%) and platelet transfusions in two. Nine patients (18%) were treated with epoetin. Granulocyte-colony stimulating factor administration, which was given only in case of G4 neutropaenia accompanied with fever or persisting >7 days, was necessary for eight patients (16%), and two of them were hospitalised.

The main non-haematological toxicities are summarised in Table 4. No grade 4 non-haematological toxicity was observed. Neuropathy occurred in 14 patients, but in only four of them (7%) symptoms were severe enough to compromise the activities of daily living and only one patient required discontinuation of treatment. Alopecia was not evaluated as most of the patients presented with pre-existing alopecia at enrolment because of earlier treatments. There were no unexpected non-haematological toxicities.

## Discussion

The identification of new drug combinations is a major challenge to improve the anti-tumour activity and toxicity profile of chemotherapy in patients with advanced tumours. This phase II study shows that the combination of GE and OXA is active in earlier treated patients with advanced ovarian cancer refractory to platinum–taxane administration, and achieves an overall response rate of 37% (95% CI, 24–52%). These results are encouraging when compared with other chemotherapeutic agents, that is, liposomal pegylated doxorubicin or topotecan and weekly paclitaxel, or GE used alone, which achieve objective overall response rates of only 10–20% in clinical trials conducted in the same setting (Gordon *et al*, 2001; Markman *et al*, 2006; Ferrandina *et al*, 2008).

The regimen used in the present trial is derived from the phase I study conducted by Mavroudis *et al* in patients with advanced solid tumours with increasing doses of GE 1000–1600 mg m^–2^ on days 1 and 8, combined with OXA 60–120 mg m^–2^ on day 8, repeated every 21 days. The dose-limiting toxicities were grade 3–4 neutropaenia, thrombocytopaenia and asthaenia (Mavroudis *et al*, 2000). The doses of GE and OXA used in our study were 25% lower than those recommended in the phase I study by Mavroudis *et al* to account for the increased toxicity of chemotherapy in the subset of refractory or resistant ovarian cancer patients. The regimen was remarkably well tolerated by our patients regarding haematological, digestive, and renal toxicities, the major toxic reactions being neutropaenia and peripheral neuropathy. The once every 3 weeks schedule of administration allowed to maintain treatment dose intensity, with 93% of planned cycles effectively administered at the planned dose. The main cause of treatment delay was haematological toxicity. Cumulative non-haematological toxicities were asthaenia and paraesthesia, as earlier reported with GE and OXA, respectively (Mavroudis *et al*, 2000; Faivre *et al*, 2002). No bleeding and rare sepsis episodes (3%) were observed, and only two patients required platelet transfusion. Digestive toxicity was easily manageable with classical anti-emetics. As OXA has no renal toxicity, treatment could be given on an out-patient basis.

Other schedules of the GE–OXA combination have been tested in phase I (Mavroudis *et al*, 2000; Gandara *et al*, 2001; Faivre *et al*, 2002) and phase II trials in different diseases and lines of treatment (Table 5). Two main schedules have been reported: concurrent administration of GE and OXA once every 2 weeks or once every 3 weeks (with GE given at days 1 and 8). If grade 3–4 haematological toxicity seems slightly more frequent with the 3-weekly regimen (>40% *vs* <20%), grade 2–3 peripheral neuropathy is clearly more frequent with the every 2-week schedule (>20% *vs* <10%), giving the opportunity to the physician and the patient to choose the more appropriate schedule as a function of earlier toxicity and late adverse effects of earlier treatments.

The combination of GE and OXA has been reported to show activity in ovarian cancer both in earlier treated (Faivre *et al*, 2002; Raspagliesi *et al*, 2004; Germano *et al*, 2007; Harnett *et al*, 2007) and in first-line treated (Steer *et al*, 2006) patients. Platinum-resistant or platinum-refractory patients treated with the OXA–GE combination have been reported to experience response rates between 20 and 26% (Raspagliesi *et al*, 2004; Harnett *et al*, 2007) with median PFS and OS of 5.0 and 9.2 months, respectively. (Raspagliesi *et al*, 2004; Harnett *et al*, 2007). The results reported in the present study fall in the same range as those of earlier reports.

The last, but not least, question to be discussed is the benefit of the combination over single-agent therapy for this subset of very poor prognosis patients. Our non-randomised study cannot provide a clear-cut answer to this fundamental question. Several studies in resistant ovarian cancer patients have failed to find a median PFS or OS advantage of combinations of doxorubicin or epirubicin with paclitaxel over paclitaxel alone, or of doublets including topotecan over topotecan alone, suggesting that non-platinum single-agent therapy might be the most appropriate treatment in this setting (Bolis *et al*, 1999; Buda *et al*, 2004; Sehouli *et al*, 2008). The median time to progression of 4.6 months and the median OS of 11.4 months in resistant or refractory patients treated with OXA and GE in our study also appear comparable to survival durations reported in earlier trials of liposomal pegylated doxorubicin, topotecan or weekly paclitaxel used as single agents (Gordon *et al*, 2001; Markman *et al*, 2006). However, median PFS and OS, in contrast to disease control and quality of life, may not be the most relevant end-points for trials conducted in patients with refractory or resistant ovarian cancer. If we consider the entire study population, the GE–OXA combination induced response or stable disease in 63% of the cases, allowing prolonged disease control with acceptable side effects in a majority of patients. The high response rate reported with the GE–OXA regimen in our patients as well as in other studies might be of value in a selected population of treatment-resistant patients who complain of symptoms and need rapid relief. This should be confirmed in more adapted trials in which long-term improvement of symptoms could be a relevant end-point to compare the benefits of combination with those of single-agent therapy. The GE–OXA combination has shown significant response rates, both in the nine patients with TFI between 0 and 3 months (44% overall response rate) and in the 33 patients who relapsed between 3 and 6 months after treatement (42% response rate), thus encouraging its use in patients with symptomatic disease. This high activity of OXA–GE should, however, be balanced against the increased toxicity of the combination compared with single-drug regimens. A comparison of the duration of disease control and patient quality of life achieved with OXA–GE or non-platinum agents used as single agents is warranted.

Finally, our multicentre experience of the OXA–GE combination in patients with resistant ovarian cancer is close to that reported in other types of platinum-refractory tumours (Faivre *et al*, 2002; Franciosi *et al*, 2003; Pectasides *et al*, 2004). Despite experimental (Bergman *et al*, 1996) and clinical evidence of synergism between these two drugs, their optimal administration, either sequential or concurrent, remains to be determined in resistant ovarian cancer patients.

## Figures and Tables

**Table 1:** Patient characteristics (*n*=50)

**Characteristics**	**%**	***n***
*Age (years)*		
Median		64
Range		46–79
		
*Histological type*		
Serous	78	39
Endometrioid	4	2
Others	18	9
		
*Histological grade*		
1	16	8
2	28	14
3	26	13
Unknown	30	15
		
*Number of earlier chemotherapy regimens*		
One	84	42
Two	16	8
		
*Response to earlier platinum–taxane regimen*		
Clinical complete	42	21
Clinical partial	26	13
Stabilisation	16	8
Progression	10	5
Unknown	6	3
		
*Performance status*		
0	32	16
1	68	34
		
Ascites	48	24
		
*Measurable lesion*		
<5 cm	40	20
⩾5 cm	10	5
No (CA 125 ⩾40 UI ml^–1^)	46	23
Not evaluable	4	2
		
*Number of disease sites* a		
1	55	27
>1	45	23

a Peritoneal or retroperitoneal or single visceral disease.

**Table 2:** Response rates

**Response evaluation**	**Measurable disease no. (%)**	**CA 125 assessable no. (%)**	**Total no. (%)**
Overall response	8 (31)	10 (43)	18 (37)
Stable disease	8 (31)	5 (22)	13 (27)
Progression	10(38)	6 (26)	16 (33)
Not evaluable	—	2 (9)	2 (3)
			
Total	26 (100)	23 (100)	49 (100)

**Table 3:** Worst haematological toxicities, and treatments

	**NCI—CTC grade 3–4**	
	**% Cycles**	**% Patients**
Leucopaenia	20	27
Neutropaenia	33	51
Thrombocytopaenia	19	26
Anaemia	8	12
Febrile neutropaenia	3	3
		
G-CSF use	14	16
EPO use	15	18
RBC transfusion	6	12
Platelet transfusion	6	3

Abbreviations: EPO=erythropoietin; G-CSF=granulocyte-colony stimulating factor; NCI—CTC=National Cancer Institute—Common Toxicity Criteria; RBC=red blood cells.

**Table 4:** Non-haematological toxicities

	**NCI—CTC grade (% patients)**			
**Toxicity**	**1**	**2**	**3**	**4**
Nausea or vomiting	28	25	7	—
Mucositis	10	8	4	—
Constipation	11	12	—	—
Diarrhoea	9	3	—	—
Infection	5	11	2	—
Neuropathy	21	6	1	—
Asthaenia	26	33	8	
Hypersensitivity	0	0	2	

Abbreviation: NCI—CTC=National Cancer Institute—Common Toxicity Criteria.

**Table 5:** Published studies with gemcitabine and oxaliplatin in cancer patients

**Reference**	**Ph**	**No. of patients**	**Disease**	**Schedule (mg m^−2^)**	**Lines of CT**	**% OR**	**MTD DLT**	**Grade 3–4 haematotox**	**Grade 2–3 non-haematotox**
*All tumours*									
Mavroudis *et al* (2000)	I	48	All	O: 120 D1 G: 100–1600 D1, D8 Every 3 w	35% 1L 23% 2L 42%⩾3L	13	G:1600 O:120	9% PNN 5% PLT 1% Hb	29% asthaenia 9% vomiting 7% oedema 4% diarrhoea
							No DLT		6% neuroT
Gandara *et al* (2001)	I	21	All	O: 130 D1 G: 1250 D1D8 Every 3 w	100% ⩽4	0	G: 1000 O: 130 DLT: PLT and confusion	ND	ND
Faivre *et al* (2002)	I/II	44	35 pulm 9 Ov	O: 70–100 D1 G: 800–1600 D1 Every 2 w	69% 1L 20% 2L 11% 3L	P 33 Ov 33	G: 1500 O: 85 No DLT	20% PNN 9% PLT	45% asthaenia 39% vomiting 43% neuroT
Franciosi *et al* (2003)	II	32	Pulm	O: 85 D1, D8 G: 1000 D1, D8 Every 3 w	75% 1 L 25% 2L	16		13% PNN 23% PLT	3% vomiting 9% hepatic 6% diarrhoea 9% neuroT
Pectasides *et al* (2004)	II	26	Germ cell	O: 130 D1 G: 1000 D1, D8 Every 3 w	62% 2L 38% 3L	32		62% PNN 41% PLT	27% vomiting 17% asthaenia 3% diarrhoea 10% neuroT
Theodore *et al* (2006)	II	30	TCC	O: 85 D1 G: 1500 D1 Every 2 w	100% 1L	47		10% PNN 2% PLT	80% Asthaenia 24% Vomiting 39% neuroT
Louvet *et al* (2002)	II	64	Pancreas	O: 100 D2 G: 1000 D1 Every 2 w	100% 1L	30		11% PNN 11% PLT	14% asthaenia 14% vomiting 11% neuroT
									
*Ovarian T only*									
Raspagliesi *et al* (2004)	II	20	Ov	O: 130 D8 G: 1000 D1D8 Every 3 w	100% 2L	26		40% PNN 70% PLT 15% Hb	45% vomiting 20% neuroT 15% hepatic 10% allergic
Steer *et al* (2006)	II	20	Ov	O: 130 D8 G: 1250 D1D8 Every 3 w	100% 1L	80		25% PNN 5% Hb	15% asthaenia 5% hepatic 5% diarrhoea 10% vomiting 20% neuroT
Germano *et al* (2007)	II	21	Ov	O:100 D2 G: 1000 D1 Every 2 w	50% 2L 50% 3L	23		30% PLT	Grade I-II: 38% nausea 38% neuroT
Harnett *et al* (2007)	II	75	Ov	O: 130 D8 G: 1000 D1D8 Every 3 w	100% 2L	20		61% PNN 10% PLT	16% nausea 22% vomiting 9% neuroT 7% dyspnoea
Present study	II	50	Ov	O:100 D1 G: 1000 D1D8 Every 3 w	84% 2L 16% 3L	37		51% PNN 26% PLT 12% Hb	32% nausea 41% asthaenia 3% diarrhoea 3% allergic 7% neuroT

Abbreviations: CT=chemotherapy; D=day; DLT=dose-limiting toxicity; G=gemcitabine; hemato tox=haematological toxicity; L=line; MDT=maximal dose tolerated; neuroT=neurotoxicity; O=oxaliplatin; OR=objective response; Ov=ovarian cancer; P=pulmonary; Ph=phase; PLT=platelet; PLT=platelet; PNN=polynuclear neutrophil; TCC=transitional cell carcinoma; w=weeks.
